# For pet’s sake: Discovering a naturally occurring zebrafish virus

**DOI:** 10.1371/journal.pbio.3002643

**Published:** 2024-06-10

**Authors:** Mollie Virgo, Brian Thomas Ho, Serge Mostowy

**Affiliations:** 1 Institute of Structural and Molecular Biology, Department of Biological Sciences, Birkbeck College, London, United Kingdom; 2 Department of Infection Biology, London School of Hygiene and Tropical Medicine, London, United Kingdom; 3 Institute of Structural and Molecular Biology, Division of Biosciences, University College London, London, United Kingdom

## Abstract

Zebrafish are often used to model host-pathogen interactions, but few models of natural virus infection have been established. This Primer explores a new study in PLOS Biology that presents a strategy to uncover viruses that are natural pathogens of laboratory zebrafish using meta-transcriptomics and co-housing experiments.

## Teaser

Zebrafish are often used to model host-pathogen interactions, but few models of natural virus infection have been established. A new study in PLOS Biology shows that metatranscriptomics and cohousing experiments can uncover a natural pathogenic virus of zebrafish for laboratory study.

Zebrafish (*Danio rerio*) have emerged as an important nonmammalian vertebrate model to study infection biology [1]. First used to study *Escherichia coli* injections [2], laboratory zebrafish are best known for modelling host–bacteria interactions in vivo using the natural fish pathogen *Mycobacterium marinum* [3]. Zebrafish models have also been established for viral infections, including some human viruses such as Sindbis, Chinkingunya, and Norovirus [4–6]. However, viral host range is often a limiting factor for successful zebrafish infection, as exemplified in the case of SARS-CoV-2 [7]. As such, despite zebrafish possessing an immune system that is highly homologous to that of humans with orthologs of classical inflammatory cytokines (such as IL1β, TNFα), type I interferons (IFNs), and interferon-stimulated genes (ISGs) [8], their use in studying antiviral defence mechanisms has been limited by the need to discover viruses naturally associated with zebrafish. Initial attempts to identify native zebrafish viruses were performed by Balla and colleagues by generating transgenic zebrafish that expressed green fluorescent protein (GFP) in response to IFN release, which led to the detection of a previously unknown zebrafish picornavirus (ZfPV) [9]. In this issue of *PLOS Biology*, Rice and colleagues significantly advance this research vision, presenting an innovative pipeline to detect and characterise natural host–virus interactions [10].

**Fig 1:**
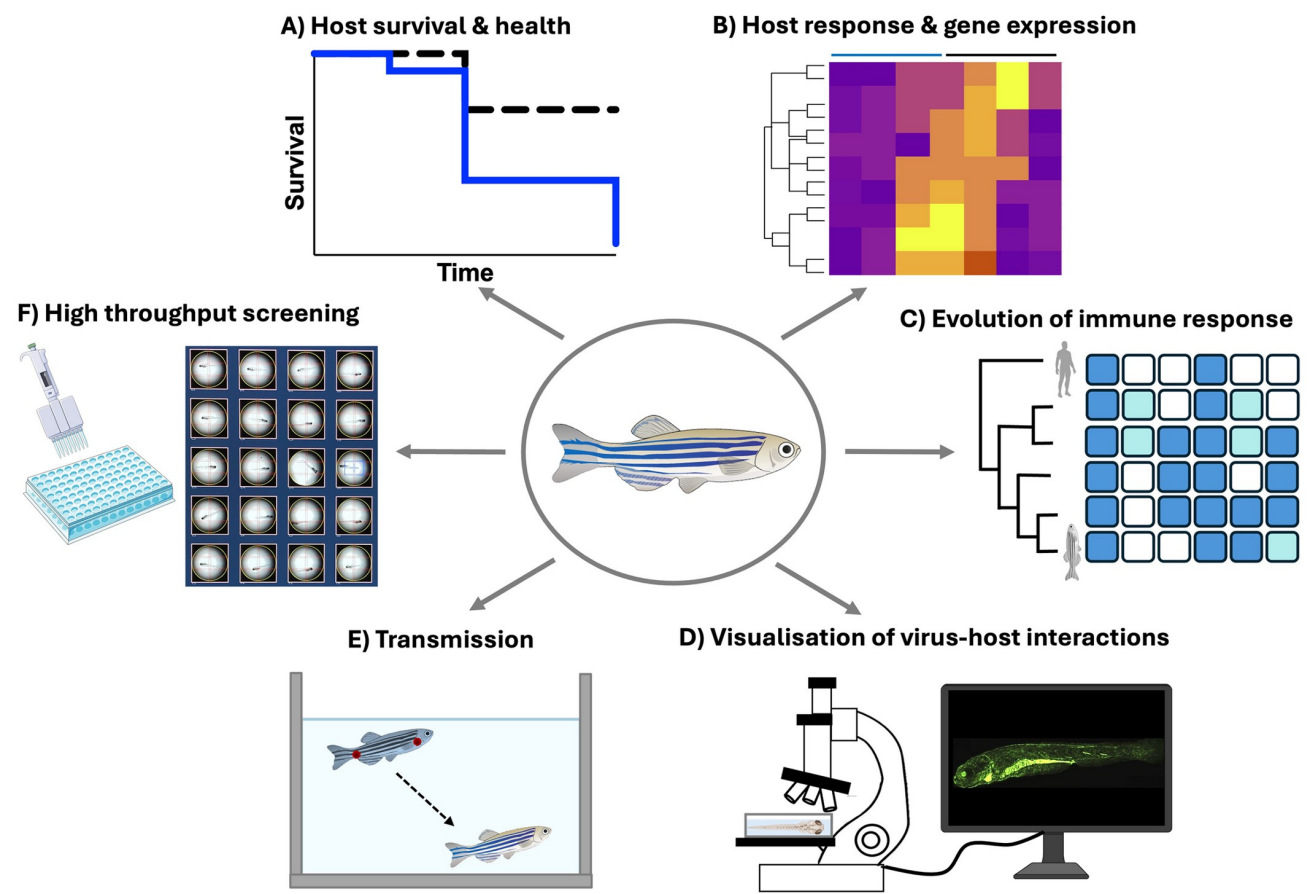
Experimental approaches to study zebrafish–virus interactions for fundamental and translational advance. (**A**) Tracking host survival and health can inform infection severity and disease phenotypes. (**B**) Characterisation of host response and gene expression (using RNA-sequencing or qRT-PCR) can illuminate mechanisms underlying antiviral immunity and inflammation. (**C**) Studying evolution of immune response can inform fundamental determinants of host defence. (**D**) Visualisation of host–virus interactions using high-resolution microscopy techniques can illuminate antiviral response from the level of the single cell to the whole animal (induced GFP expression in transgenic zebrafish reporting on IFN signalling adapted from [9]). (**E**) Investigation of transmission to uncover host/viral determinants that foster transmission and improve colony health monitoring programs. (**F**) High-throughput screening of pharmacological compounds and host factors can identify therapeutic approaches and enable translational advance. GFP, green fluorescent protein; IFN, interferon; qRT-PCR, quantitative reverse transcription PCR.

The study shows that metatranscriptomic sequencing and cohousing experiments can be used to discover zebrafish-associated microbes [10]. Identified from pet trade zebrafish, the authors report Rocky Mountain birnavirus (RMBV) as a natural zebrafish virus, which is transmissible and pathogenic to laboratory reared zebrafish. As a resource, the pipeline presented here establishes a paradigm for the discovery of unknown infectious agents. More broadly, a greater appreciation for zebrafish viruses can inspire the use of zebrafish to study antiviral immunity and transmission dynamics, and transform our understanding of host–pathogen interactions (**Fig 1**).

Technologies such as 16S rRNA sequencing and histopathology are limited when it comes to detecting unknown microbes associated with zebrafish. To address this, the authors employed a state-of-the-art metatranscriptomic survey to compare microbes associated with zebrafish in pet trade versus laboratory environments. Unlike pet trade zebrafish, laboratory zebrafish are reared within a highly controlled environment with routine health monitoring, regulated feeds, and periods of quarantine for new zebrafish (before mixing with the wider colony). In their analysis, a number of potential pathogens were identified in pet trade zebrafish, which were absent in laboratory-reared zebrafish. Most striking was the discovery of a newly described RNA virus, RMBV, in pet trade zebrafish. Following genome reconstruction and phylogenetic analysis, RMBV was found to belong to a clade of viruses that naturally infect commercially important fish species (such as trout and salmon). Even though RMBV carrying pet trade zebrafish lacked any visible disease phenotype, they expressed high levels of ISGs compared to laboratory-reared zebrafish, pointing to an RMBV-associated antiviral defence response. Considering this, the induction of ISGs as a transcriptional phenotype is highly beneficial for researchers, allowing for detection of otherwise undetectable host–virus interactions [9].

Having discovered a virus naturally associated to zebrafish, the authors next sought to determine whether RMBV could be transmitted to laboratory zebrafish. In an elegant set of experiments, the authors cohoused pet trade and laboratory zebrafish and monitored health. One month after cohousing, haemorrhaging was observed in 3/10 of the laboratory zebrafish, which correlated with high levels of RMBV in multiple anatomical sites (kidney, spleen, intestine), indicating systemic infection. This demonstrates that circulating RMBV in outwardly healthy pet trade zebrafish can be naturally transmitted to laboratory zebrafish, causing haemorrhagic disease. The RMBV-induced haemorrhaging in a subset of laboratory zebrafish is particularly remarkable given the asymptomatic carriage in pet trade zebrafish. Demonstrating causation remains challenging when moving from pet store to lab-reared animals. Genetic background, immunological life history, host microbiome, and stage of infection could all be contributing to differences in RBMV susceptibility and infection outcome. To begin to decipher the spectrum of disease states following RMBV exposure, the authors performed a second cohousing experiment. Surprisingly, no transmission to laboratory zebrafish was detected and no haemorrhaging was observed under these experimental conditions (0/10). These clever experiments show that zebrafish can be carriers of RMBV without transmitting it, although future work will be required to decipher the mechanisms and dynamics underlying RMBV transmission to help control its spread for improved colony health.

The authors further characterised the host transcriptional response to RMBV infection by comparing the gene expression of infected laboratory zebrafish to that of control laboratory zebrafish. Over 800 genes were identified as being significantly up-regulated in RMBV-infected zebrafish, including IFN signalling genes such as isg15, a key player in the host antiviral response and useful readout of IFN release [9]. To better appreciate the haemorrhagic inflammatory response, the authors compared their data set with another publicly available data set that captured gene expression following infection of zebrafish with spring viremia of carp virus (SVCV). SVCV is an RNA virus that also causes haemorrhagic disease in zebrafish. Significant overlap in genes up-regulated following RMBV and SVCV exposure was observed, highlighting the use of RMBV zebrafish infection as a model to explore fundamental aspects of host–virus interactions. Identifying molecular signatures common among viruses inducing haemorrhagic disease has great potential to deliver biomarkers for virus discovery and novel insights regarding the evolution of host–virus interactions.

In the same way that zebrafish infection using *M*. *marinum* has become a paradigm for studying natural host–bacteria interactions in vivo, one can imagine that zebrafish–RMBV interactions may similarly inspire infection biologists. While research using zebrafish has traditionally explored host transcriptional responses to experimentally administered viruses, few studies have tracked host transcriptional responses during infections by naturally occurring viruses in any vertebrate system [9]. The research pipeline presented here to detect natural virus infections of zebrafish, and the discovery of RMBV, can promote both fundamental and translational advance. In the future, it will be exciting to exploit the optical transparency of zebrafish to visualise host–RMBV interactions and take advantage of innovative microscopy approaches for high content screening of compounds or host factors to manipulate the infection process. A better understanding of zebrafish–virus interactions will reveal evolutionarily conserved mechanisms of antiviral immunity and propel the use of zebrafish for infection biology research (Fig 1). In this way, zebrafish infection models can be better exploited to illuminate new possibilities to control future emerging threats in aquaculture and society.

## Abbreviations

GFP: green fluorescent protein
IFN: interferon
ISG: interferon-stimulated gene
RMBV: Rocky Mountain birnavirus
SVCV: spring viremia of carp virus
ZfPV: zebrafish picornavirus
